# An Update of Phenotypic–Genotypic IMNEPD Cases and a Bioinformatics Analysis of the New *PTRH2* Gene Variants

**DOI:** 10.3390/genes15121508

**Published:** 2024-11-25

**Authors:** Rajech Sharkia, Marie-Laure Vuillaume, Sahil Jain, Muhammad Mahajnah, Radka Stoeva, Agnès Guichet, Estelle Colin, Jérome Champ, Nicolas Derive, Arnaud Chefdor, Abdelnaser Zalan

**Affiliations:** 1 Unit of Human Biology and Genetics, The Triangle Regional Research and Development Center, Kafr Qari 3007500, Israel; dr.zalan@hotmail.com; 2 Unit of Natural Sciences, Beit-Berl Academic College, Beit-Berl 4490500, Israel; 3 Genetics Department, Tours University Hospital, 37044 Tours, France; 4 INSERM, Imaging Brain & Neuropsychiatry iBraiN U12523, University of Tours, 37032 Tours, France; 5 Bioinformatics Centre, Dr. D.Y. Patil Biotechnology and Bioinformatics Institute, Dr. D.Y. Patil Vidyapeeth, Pune 411033, India; 6 The Ruth and Bruce Rappaport Faculty of Medicine, Technion-Israel Institute of Technology, Haifa 3109600, Israel; 7 Child Neurology and Development Center, Hillel Yaffe Medical Center, Hadera 3810000, Israel; 8 Department of Medical Genetics, Le Mans Hospital, 72037 Le Mans, France; 9 Genetics Department CHU 4 Rue Larrey, 49933 Angers, France; 10 Miotvasc, UMR CNRS 6015, INSERM U1083, Angers University, 49933 Angers, France; 11 SeqOIA Laboratory, FMG2025, 75014 Paris, France; 12 Department of Pediatrics, Le Mans Hospital, 72037 Le Mans, France

**Keywords:** *PTRH2* gene, PTRH2 variants, IMNEPD, clinical features, rare genetic diseases, autosomal recessive disorder, bioinformatics analysis

## Abstract

Background/Objectives: Biallelic mutations in the *PTRH2* gene are associated with a rare genetic disease known as infantile-onset multisystem neurologic, endocrine, and pancreatic disease (IMNEPD). In this study, we describe a new case carrying a previously identified mutation, provide an updated analysis of the relative frequencies of the clinical features across all published cases (including the three latest studies), and perform a bioinformatics analysis of the newly identified PTRH2 protein variants from a structural perspective. Methods: Clinical examination of the patients was carried out, and genetic testing was performed using a genome sequencing strategy. A bioinformatics analysis was carried out for the newly reported mutations using PYMOL that was utilized to view the structure and analyze the mutations. Additionally, the ThermoMPNN webserver was employed to check the effect of point mutations on the overall stability of the protein. Results: Our findings indicate that motor delay, neuropathy, intellectual disability, distal weakness, hearing impairment, and ataxia are the most common symptoms, while the other clinical features fall into two frequency categories: moderately common ones and the least common ones. The bioinformatics analysis revealed that the Q85 residue is highly conserved, suggesting that mutations at this position could disrupt key signaling pathways or cellular functions. Indeed, the Q85R mutation was shown to significantly impair the stability and functionality of the protein. Conclusions: The clinical presentation of IMNEPD remains highly variable in terms of both severity and progression. Mutations at the Q85 residue have been identified in nearly 50% of reported cases, highlighting this position as a potential mutational hotspot in the PTRH2 protein.

## 1. Introduction

The disease abbreviated as IMNEPD (infantile-onset multisystem neurologic, endocrine, and pancreatic disease) is considered to be a rare autosomal recessive genetic disorder. It was first reported in two patients as described by Hu and colleagues in the year 2014 [1]. The causes of this disorder were found to be biallelic mutations in the peptidyl-tRNA hydrolase 2 (*PTRH2*) gene located on chromosome 17 (NG_042064.1) [2,3]. The PTRH2 protein, which is also referred to as BIT1 (Bcl-2 inhibitor of transcription 1), is a highly conserved protein that belongs to the peptidyl-tRNA hydrolase family. It functions by releasing the peptidyl component from tRNA, thereby preventing the accumulation of prematurely dissociated peptidyl-tRNA, which can hinder protein synthesis and be harmful to cells. Additionally, PTRH2 plays a crucial role in regulating cell survival and apoptosis, as well as influencing muscle differentiation during human development; therefore, mutations in the *PTRH2* gene that impair its function can lead to congenital IMNEPD [4,5,6]. Such mutations can result in a reduction in or complete loss of the PTRH2 protein’s activity, potentially disrupting mitochondrial translation and contributing to IMNEPD. Previous studies have shown that missense and nonsense mutations in the *PTRH2* gene can result in cases of IMNEPD with varying degrees of severity [7,8,9].

As IMNEPD is known to be a multisystem disorder, it exhibits many varying clinical features. These phenotypic characteristics include global developmental delay, intellectual disability, sensorineural hearing loss, ataxia, pancreatic insufficiency, postnatal microcephaly, peripheral neuropathy, facial dysmorphism, cerebellar atrophy, hypothyroidism, diabetes mellitus, and liver dysfunction [3,10,11,12].

We previously conducted an extensive study of all cases of IMNEPD and the various *PTRH2* gene variants involved, as well as their phenotypic characteristics, in addition to a bioinformatics analysis [8]. A later study from Saudi Arabia reported two siblings with a homozygous mutation in the *PTRH2* gene, namely c.114dup, p.(Gly39Trpfs*16) [13]. The most recent study from Bahrain [14] reported two siblings suffering from IMNEPD carrying a homozygous mutation in the *PTRH2* gene NM_016077.5:c.370del p.(Glu124Lysfs*4). Furthermore, another study in the same year reported two sisters of Iranian origin who had a homozygous pathogenic variant NM_016077.5:c.254A>G (p.Gln85Arg) in the *PTRH2* gene [9].

Here, in the current study, we report a consanguineous family in which two siblings carry a homozygous mutation NM_016077.5:c.269_270delCT, (p.Ala90Glyfs*13) in the *PTRH2* gene, which is similar to the variant that was previously reported by Hu and coworkers [1]. The main clinical manifestations resemble those of the previously described cases but expand the clinical phenotype to include additional, unreported features, which are self-mutilative behavior, picky eating, and sleep disturbances. Further, we update the presentation of the relative frequencies of various clinical manifestations of all published cases, including the three new studies conducted in the current year, in addition to our current case, which involved two patients. Additionally, a bioinformatics analysis was carried out for the newly reported mutations in the *PTRH2* gene.

## 2. Methodology

In the current study, all available cases involving *PTRH2* gene variants (up until the date of preparation of this study, at the end of the year 2024) were presented, along with their clinical features (Table S1: Supplementary Data). An extensive literature review was carried out using the PubMed and Google Scholar websites (https://pubmed.ncbi.nlm.nih.gov/, accessed on 10 October 2024 and https://scholar.google.com/, accessed on 10 October 2024), with keywords used for the search such as *PTRH2* gene, PTRH2 variants, *PTRH2* mutations, NM_016077.5, infantile-onset multisystem neurologic, endocrine, and pancreatic disease (IMNEPD), IMNEPD clinical features, etc. Additionally, the relative frequencies of the different clinical features and genetic variants in all these cases were summarized (Table 1).

A quatuor-based genome sequencing strategy was applied to the two affected children and their parents and conducted on the SeqOIA platform as part of the French initiative “France Medecine Genomique 2025” (https://pfmg2025.aviesan.fr/en/, accessed on 20 November 2024). Library generation was performed using the NEBNext Ultra II End repair/A-tailing module and the ligation module (New England Biolabs, Ipswich, MA, USA) and quantified using qPCR with the NEBNext Custom 2X Library Quant Kit master mix (New England Biolabs) and the QuantStudio 6 Flex Real-Time PCR system (Life Technologies, Carlsbad, CA, USA). The genome was sequenced on a Flow Cell S4 on a NovaSeq6000 (Illumina, San Diego, CA, USA) following the manufacturer’s instructions. For the index case, the average depth of coverage was 51.9X. More than 96.4% of the targeted regions were covered at least 20 times. The DNA sequences were mapped to the reference human genome sequence (GRCh38.92fa) using BWA-MEM (v0.7.15). HaplotypeCaller from the Genome Analysis Toolkit (v4.v.7.0), ClinSV (v1.0.0), Wisecondor (v1.2.4), and ExpansionHunter were used to perform variant calling. Filtering was performed using annotations from SnpEff (v4.3t) and AnnotSV 3.0.7 in a custom bioinformatic pipeline.

In an earlier study conducted by our group, we carried out a computational analysis of the various PTRH2 variants (V23A, S43Kfs*11, Q85P, A90Gfs*13, Y94N, W108*, and E110*) [8]. Briefly, we carried out the analysis by predicting the PTRH2 structure with the help of AlphaFold [15] and predicting the evolutionary conservation of various amino acids using the ConSurf webserver [16].

In the current study, a bioinformatics analysis was carried out for the newly reported mutations (p.(Glu124Lysfs*4), p.(Gly39Trpfs*16), and p.(Gln85Arg)) [9,13,14] using our previously predicted PTRH2 structure. PYMOL [17] was utilized to view the structure and analyze the mutations. The ThermoMPNN webserver [18] was employed to check the effect of point mutations on the overall stability of the protein. ThermoMPNN is a deep-neural-network-based tool designed to predict stability changes due to point mutations in proteins. It is trained on two datasets: (a) a sequence recovery dataset used to train the ProteinMPNN program and (b) a dataset consisting of experimental stability measurements for hundreds of mutations in hundreds of proteins.

## 3. Results

### 3.1. Case Presentation

The family of Arab Syrian origin in France was referred to the pediatric neurology and genetic department of Le Mans Hospital (France) due to progressive sensorineural hearing loss, peripheral neuropathy, and gait disturbance. The parents are relatives (second cousins) and healthy with five children: three healthy children (two sisters and one brother) and two affected siblings.

The eldest affected sibling is a 19-year-old male who was born at full-term pregnancy by vaginal delivery, with normal birth parameters. Immediately after birth, no abnormal signs were noticed except for neonatal hypotonia. He started walking at the age of 1 year and 7 months. At the same age, he was diagnosed with congenital hypothyroidism and treated with Levothyroxine. Later, the family reported that he presented with atypical feeding (picky eating), self-mutilation behavior, sleep disturbance (treated with Melatonin and Gabapentin), and global developmental delay, including speech and language delay and motor (gait) delay.

**Figure 1:**
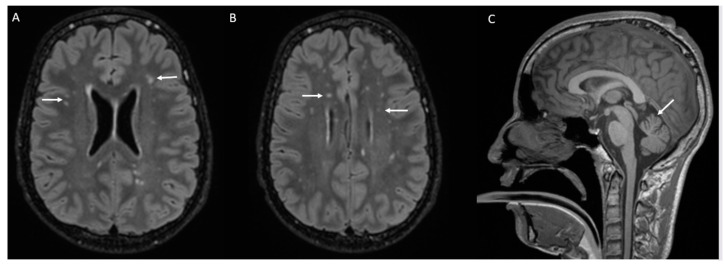
(**A**,**B**): The axial FLAIR-weighted image shows multiple small hyperintensity signals in the deep white matter, with a radial distribution around the lateral ventricles, predominantly in the bilateral fronto-parietal regions (arrow). (**C**): The sagittal T1-weighted image shows mild atrophy of the superior cerebellar vermis (arrow).

His last neurological examination at the age of 19 years old revealed normal growth parameters (weight percentile: 10; height: −2 SD; head circumference: −1 SD), four café au lait spots, mild pes cavus, gait disturbance with progressive ataxia, and severe sensory and motor peripheral neuropathy, including neuropathic pain in the upper and lower extremities. He has severe expressive and language comprehension delays. He was diagnosed with bilateral moderate progressive sensorineural hearing loss using audiometry. Recently, he was diagnosed with insulin-dependent diabetes. Brain magnetic resonance imaging (MRI) revealed multiple periventricular FLAIR signals, predominant in the fronto-parietal area, with mild vermis atrophy (Figure 1).

The second patient is a 11-year-old male who was born at full-term pregnancy by vaginal delivery (as a fourth pregnancy), with normal birth parameters. Immediately after birth, no abnormal signs were noticed except for neonatal hypotonia. At the age of 17 months, he was diagnosed with congenital hypothyroidism and treated with Levothyroxine. He started walking at the age of 1 year and 7 months. Later, the family reported that he presented with sleep disturbance (treated with Melatonin) and global developmental delay, including speech and language delay and motor (gait) delay.

His last neurological examination at the age of 11 years old revealed normal growth parameters (weight percentile: 10; height: −1 SD; head circumference: −1 SD), abnormality of the hallux, reducible talipes equinovarus (incipient), mild pes cavus, gait disturbance with progressive ataxia, and mild sensory and motor peripheral neuropathy of the upper and lower extremities. His ophthalmic examination revealed bilateral strabismus and hypermetropia. He has severe expressive and language comprehension delays. He was diagnosed with bilateral moderate progressive sensorineural hearing loss using audiometry. Brain magnetic resonance imaging (MRI) revealed multiple periventricular FLAIR signals, predominant in the fronto-parietal area, with mild vermis atrophy and enlarged perivascular spaces. He had a regular endocrine follow-up, but no diabetes was detected. He had surgery for a rectal prolapse.

As the homozygous mutation carried by our patients was the same as that described by Hu and coworkers [1], we opted to compare their respective clinical features with those from their study and present them in Table 1.

The common clinical features in both cases were similar, which were sensorineural deafness, global developmental delay, intellectual disability, ataxia, sensory and motor peripheral neuropathy, distal muscle weakness, and cerebellar atrophy. On the other hand, some clinical features were not displayed by our patients while they were present in the patients described by Hu and his coworkers. These features include deformity of the head and face, hand deformity, microcephaly, exocrine pancreas insufficiency, and liver fibrosis. Behavioral difficulties, which were self-mutilative behavior, picky eating, and sleep disturbances, were observed in our cases while they were not reported by Hu and colleagues [1].

Whole genome sequencing (WGS) was carried out for the two patients and revealed a homozygous truncating variant in *PTRH2*: NM_016077.5:c.269_270del p.(Ala90GlyfsTer13). The parents were also found to be carriers (heterozygous) of this pathogenic variant, while the other siblings were not tested genetically. This variant was absent in the GnomAD v4 population control database (while the two pathogenic variants, i.e., c.324G>A, p.Trp108* and c.127dupA, p.Ser43Lysfs*11, were present in this database), and it was reported twice as a pathogenic variant in the ClinVar database (VCV0000183428.18), as well as in the literature [1].

Since the first case of IMNEPD that was described in 2014, to date, about 32 cases have been reported. In this study, a comprehensive literature review of all these cases, along with their clinical features, was carried out, and it is presented in Table S1. The relative frequencies of various clinical features in these cases were calculated and are displayed in Table 2.

It was found that the most common clinical characteristics amongst all the patients were motor delay (~94%), neuropathy (~89%), intellectual disability (87.5%), distal weakness (85.2%), hearing impairment (78.12%), and ataxia (~77%). The moderately common characteristics included hand deformity (~58.1%), cerebellar atrophy/hypoplasia (56.5%), and deformity of the head and face (53.3%). Meanwhile, the least common characteristics were diabetes mellitus (40%), pancreatic abnormality (~33.3%), hypothyroidism (~21.9%), and liver abnormality (~17.4%).

The results revealed that the majority of the *PTRH2* gene variants detected in the 32 cases were missense mutations, with a relative frequency of about 56%, while the nonsense mutations had a relative frequency of 44% (Table 2). Four missense mutations in the *PTRH2* gene were reported. Amongst these, the most common mutation was Q85P, which was reported in four different Arab communities, as indicated in Table S1. The relative frequencies of the other three missense mutations were about 12% (Table 2).

Six different nonsense mutations were reported in the *PTRH2* gene. Four of them (W108*, E110*, G39W*16, and E124K*4) were found in one family each, while each of the other two mutations (S43K*11 and A90G*13) were reported in two unrelated families (Table S1).

### 3.2. Structural Analysis of the Mutational Effects

In our earlier study, we predicted the PTRH2 protein’s structure and designated its domains [8]. Briefly, residues 1–62 form the mitochondrial localization sequence (MLS), while residues 63–179 form the catalytic hydrolase domain [6]. Based on this information, a bioinformatic analysis was carried out for the new mutations as follows.

#### 3.2.1. Glu124Lysfs*4

Glu124Lysfs*4 is a frameshift mutation leading to the formation of a truncated PTRH2 protein without a major chunk of the catalytic hydrolase domain.

#### 3.2.2. Gly39Trpfs*16

Gly39Trpfs*16 is a frameshift mutation leading to the formation of a non-functional PTRH2 protein without part of the mitochondrial localization sequence and completely lacking the catalytic hydrolase domain.

#### 3.2.3. Gln85Arg

**Figure 2:**
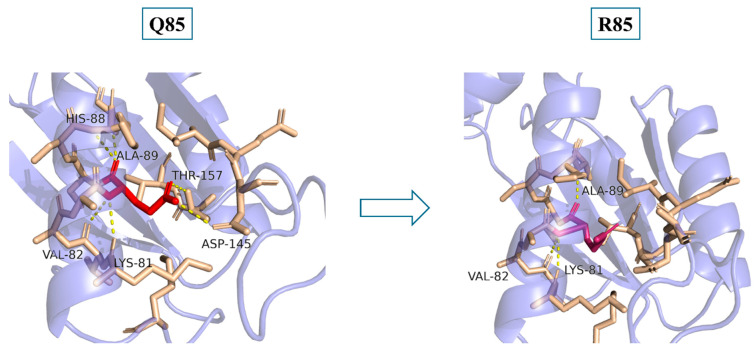
A zoom-in view of the predicted PTRH2 protein structure. The 85th residue is shown in red, while the surrounding residues are shown in brown. A strong polar network is observed in the presence of Q85 of interaction with K81, V82, H88, A89, D145, and T157 (**left panel**). However, the Q85R mutation results in the loss of many of these interactions (**right panel**), resulting in destabilization of the protein. Also, as the lost interactions include residues 85 and 157, which are part of a putative active site, the mutation probably affects the enzyme functionality.

Q85 is a highly conserved residue, as indicated by the ConSurf analysis [8]. Replacing a neutrally charged residue (glutamine) with a positively charged residue (arginine) can be expected to disrupt the local regional interactions. Indeed, as shown in Figure 2, Q85 forms hydrogen bonds with H88, D145, and T157; however, these interactions are lost where Q85 is replaced with R85. The loss of these interactions, accompanied with the fact that arginine has a longer side chain than glutamine, might affect the overall stability of the structure. Also, K81 and D145 form a salt bridge under normal conditions. However, upon mutation, the Q85-D145 interaction is lost, which might displace D145 from a favorable position for interacting with K81, resulting in the loss of this strong, stabilizing salt bridge. Indeed, the DUET (https://doi.org/10.1093/nar/gku411, accessed on 10 August 2024) and ThermoMPNN webservers [18] indicate a likely decrease in the protein’s stability by −0.622 kcal/mol and 0.4245 kcal/mol, respectively.

Further analysis of the Q85R mutation suggests that the loss of a Q85-T157 interaction due to the mutation will affect the enzyme functionality, as both residues are part of a putative active site [19]. Moreover, residues 80–99 form a helix and have been reported to be involved in interactions with various proteins [6,20]. A disruptive change at the 85th residue, resulting in the loss of interactions with H88, Q145, and T157, can be expected to derange the correct conformation of this helix and consequently might hamper such protein–protein interactions. Indeed, the alphamissense database (https://doi.org/10.1126/science.adg7492, accessed on 10 August 2024) indicates that replacement of Q85 in PTRH2 with any other amino acid is generally deleterious, with a high likeliness to be pathogenic.

## 4. Discussion

In the current study, we updated all of the previously reported phenotypic symptoms in IMNEPD cases, along with the latest three studies published in the current year, in addition to our current case, which involved two siblings of Syrian origin living in France. Additionally, we present a bioinformatic analysis of the newly found variants.

According to our current update of the relative frequencies of the various clinical characteristics of IMNEPD, it was demonstrated that the hallmarks of this disease are motor delay, neuropathy, intellectual disability, distal weakness, hearing impairment, and ataxia. On the other hand, the other clinical manifestations were found to be distributed into two ranges of frequencies: the moderately common ones—hand deformity, cerebellar atrophy/hypoplasia, and deformity of the head and face (between 53% and 58%)—and the least common ones—diabetes mellitus, pancreatic abnormality, hypothyroidism, and liver abnormality (≤40%). These analyses confirm that the severity of and variability in this disease remain widely variable.

In comparing our current cases with Hu’s cases [1], it was found that the common clinical characteristics of IMNEPD were similar, while some of the moderately common and less common features of the disease were lacking in our patients. Thus, it seems that our patients have less severe disease than those described by Hu and coworkers. It is interesting to note that some behavioral difficulties were noticed in our cases, such as self-mutilative behavior, picky eating, and sleep disturbances. These clinical features were not reported in any of the previously published studies. Therefore, we suggest that such clinical features should be taken into consideration in future investigations of IMNEPD cases.

Our current data showed that a higher rate of the *PTRH2* gene variants were missense mutations than nonsense mutations (56% to 44%). It is noteworthy that this rate was previously recorded to be 64% to 36% [8]. Overall, these results confirmed that missense mutations could be found to cause less severe phenotypes compared to nonsense mutations.

The computational analysis indicated that Q85 is a highly conserved residue, and therefore, a mutation at this residue is expected to impact key signaling pathways or cellular functions. Indeed, a Q85R mutation was found to affect the overall stability and functionality of the protein. Additionally, this mutation might impact the ability of PTRH2 to interact with other proteins, potentially disrupting key cellular processes. Interestingly, a mutation at the same position, Q85P, was reported in 14 patients belonging to four different Arab communities in earlier studies presented in five publications [2,3,8,21,22]. A report of the current mutation (Q85R) in two Iranian patients [9] further strengthens our previously proposed suggestion [8] of a common-founder affect. This also indicates that the 85th residue of the PTRH2 protein might be a hotspot for mutations. It is noticeable that the severity of and variability in the clinical features of the Q85P variant resemble those in the Q85R variant.

Our study updated the relative frequencies of the clinical features of this *PTRH2*-gene-related disease. Overall, motor delay, neuropathy, intellectual disability, distal weakness, hearing impairment, and ataxia are the main common phenotypes to IMNEPD, although other symptoms can also be associated with them. As this disorder involves different systems, it leads to a severe and large spectrum of variable symptoms and makes its diagnosis a particular challenge. Therefore, we recommend a precise genetic diagnosis of suspected mutations in the *PTRH2* gene as an indication of IMNEPD, especially in patients with a family history of consanguineous marriages.

## Figures and Tables

**Table 1:** Clinical manifestations of *PTRH2* gene mutation disease (IMNEPD) from our new cases in relation to those presented in reference [1].

	PTRH2 Mutation	Our Study		[1]	
		c.269_270delCT p.(Ala90Glyfs*13) (A90Gfs*13)		c.269_270delCT p.(Ala90Glyfs*13) (A90Gfs*13)	
Clinical Features		Patient 1 (M)	Patient 2 (M)	Patient 1 (F)	Patient 2 (M)
Sensorineural deafness		++	+++	+++	+++
Neonatal hypotonia		++	++	+	-
Global developmental delay		++	++	++	++
Motor delay		+	+	+	+
Speech and language delay		+++	++	+++	+++
Intellectual disability		+	+	+	+
Deformity of the head and face		-	-	+	+
Hand deformity		-	-	+	+
**Feet abnormalities:**					
Abnormality of the hallux		-	+	+	-
Talipes equinovalgus (incipient)		-	-	+	-
Achilles tendon contracture		+++	+	+	-
Mild pes cavus		+	+	-	-
Clubfoot		-	-	-	-
Spasticity		-	-	-	-
Ataxia		+	+	++	++
Sensory and motor peripheral neuropathy		+++	++	++	++
Insensitivity to pain Neuropathic pain		-	-	-	-
		+	-	-	-
**Vision/eyes impairment:**					
Strabismus		-	+	+	+
Hypermetropia		-	+	NR ^a^	NR
Hypertelorism		-	-	+	+
**Behavioral difficulties:**					
Self-mutilative behaviour		+	-	NR	NR
Picky eating		+	-	NR	NR
Sleep disturbances		+	+	NR	NR
Microcephaly		-	-	++	++
**Genitourinary**: External genitalia		-	-	NR	Shawl scrotum
Growth delay		height − 2 SD	-	+	+
Distal muscle weakness		+	+	+	+
Exocrine pancreas insufficiency		-	-	+	+
Diabetes mellitus		+	-	+	+
Hypothyroidism		+	+	+	+
Liver fibrosis		-	-	+	+
**Brain MRI:**					
Cerebellar atrophy		+	+	+	+
Multiple periventricular FLAIR signals		+	+	-	-

^a^ NR: not reported.

**Table 2:** Frequency of various clinical and genetic variables in the reported IMNEPD cases.

Factors	Relative Frequency (N = 32)
Total number of reported families	18
Number of patients affected by the following:	
**Missense mutations:**	18 (56.25%)
(A) c.254A>C, p.Gln85Pro	14 (43.75%)
(B) c.68T>C, p.Val23Ala	1 (3.125%)
(C) c.280T>A, p.Tyr94Asn	1 (3.125%)
(D) c.254A>G, p.Gln85Arg	2 (6.25%)
**Nonsense mutations:**	14 (43.75%)
1. Nonsense nucleotide deletion (c.269_270delCT, p.Ala90Glyfs*13, c.370del, p.Glu124Lysfs*4)	5 (15.6%)
2. Nonsense point mutation (c.324G>A, p.Trp108*, c.328G>T, p.Glu110*)	4 (12.5%)
3. Nonsense nucleotide duplication (c.127dupA, p.Ser43Lysfs*11, c.114dup, p.Gly39Trpfs*16)	5 (15.6%)
Motor delay	30/32 (93.75%)
Intellectual disability	28/32 (87.5%)
Hearing impairment	25/32 (78.12%)
Deformity of the head and face	16/30 ^a^ (53.33%)
Hand deformity	18/31 (58.06%)
Distal weakness	23/27 (85.2%)
Ataxia	20/26 (76.92%)
Cerebellar atrophy/hypoplasia	13/23 (56.5%)
Neuropathy	24/27 (88.9%)
Liver abnormality	4/23 (17.39%)
Pancreatic abnormality	9/27 (33.33%)
Hypothyroidism	7/32 (21.88%)
Diabetes mellitus	12/30 (40%)

^a^ If a specific clinical feature was not mentioned for a particular patient in an article, this patient was excluded from the analysis, resulting in a reduction in the total number of patients.

## Data Availability

The original contributions presented in the study are included in the article/Supplementary Materials, further inquiries can be directed to the corresponding author.

## Supplementary Materials

The following supporting information can be downloaded at https://www.mdpi.com/article/10.3390/genes15121508/s1. Table S1: Phenotypic characteristics and *PTRH2* gene variants of all the IMNEPD published cases including our current case [23,24].
