# Supplementary material for: An Update of Phenotypic–Genotypic IMNEPD Cases and a Bioinformatics Analysis of the New PTRH2 Gene Variants
Source: Genes (Basel). 2024 Nov 25;15(12):1508. doi: 10.3390/genes15121508 (PMC11675358; doi:10.3390/genes15121508)
Supplement: Supplementary file 1 [file genes-15-01508-s001.zip › genes-3303689-supplementary.pdf]

Table S1: Phenotypic characteristics and *PTRH2* gene variants of all the IMNEPD published cases including our current case.

**Mutation Type**	**Missense Mutations**								**Nonsense Mutations**							
PTRH2 variant [a]	c.254A>C					c.68T>C	c.280T>A	c.254A>G	c.269_270delCT		c.324G>A	c.127dupA		c.328G>T	c.114dup	c.370del
PTRH2 protein variant	p.(Gln85Pro) **(Q85P)**					p.(Val23Ala) **(V23A)**	p.(Tyr94Asn) **(Y94N)**	p.(Gln85Arg) **(Q85R)**	p.(Ala90Glyfs*13) **(A90Gfs*13)**		p.(Trp108*) **(W108*)**	p.(Ser43Lysfs*11) **(S43Kfs*11)**		p.(Glu110*) **(E110*)**	p.(Gly39Trpfs*16) **(G39Wfs*16)**	p.(Glu124Lysfs*4) **(E124Kfs*4)**
Reference	[21]	[2]	[3]	[22]	[8]	[23]	[7]	[9]	[1]	Current case	[10]	[11]	[24]	[12]	[13]	[14]
Ethnicity	Arab	Arab	Arab	Arab	Arab	Iranian	Japanese	Irani	Turkish	Arab	Arab	Indian	NR [b]	Indian	Saudi	Bahraini
Number of patients	1	5	3	4	1	1	1	2	2	2	3	1	2	1	2	1
**Clinical Features**																
Motor delay	14/14					1/1	0/1	2/2	2/2	2/2	3/3	0/1	2/2	1/1	2/2	1/1
Intellectual disability	11/14					0/1	1/1	2/2	2/2	2/2	3/3	1/1	2/2	1/1	2/2	1/1
Hearing impairment	10/14					0/1	1/1	2/2	2/2	2/2	1/3	1/1	2/2	1/1	2/2	1/1
Deformity of head and face	10/14					0/1	1/1	0/2	2/2	0/2	2/3	0/1	NR	0/1	0/2	1/1
Hand deformity	10/14					0/1	1/1	NR	2/2	0/2	1/3	0/1	2/2	0/1	2/2	0/1
Distal weakness	11/13 [c]					1/1	1/1	2/2	2/2	2/2	2/3	0/1	NR	1/1	NR	1/1
Ataxia	6/9					NR	1/1	2/2	2/2	2/2	3/3	0/1	2/2	1/1	0/2	1/1
Cerebellar atrophy/ hypoplasia	2/9					NR	1/1	2/2	2/2	2/2	2/3	0/1	NR	1/1	1/1	0/1
Neuropathy	8/9					0/1	1/1	2/2	2/2	1/2	3/3	1/1	2/2	1/1	2/2	1/1
Liver abnormality	1/9					0/1	0/1	0/1 [c]	2/2	0/2	0/3	0/1	NR	1/1	NR	0/1
Pancreatic abnormality	1/9					0/1	1/1	1/1	1/2	0/2	2/3	0/1	1/2	1/1	0/2	1/1
Hypothyroidism	0/14					0/1	1/1	1/2	2/2	2/2	0/3	0/1	1/2	0/1	0/2	0/1
Diabetes mellitus	0/13					NR	0/1	2/2	2/2	1/2	2/3	1/1	1/2	1/1	1/2	1/1

[a] All the variants were homozygous
[b] NR: not reported
[c] When a particular clinical feature is not mentioned in the article for that specific patient, then that patient is excluded from the rest of the patients, therefore, the total number of patients will be reduced.
